# Retinal Microvascular and Structural Findings in Children with Peripheral Arteriovenous Malformations Receiving Systemic Bevacizumab: An OCTA-Based Case–Control Study

**DOI:** 10.3390/jcm15218450

**Published:** 2026-10-30

**Authors:** Burcu Gorgulu Sahin, Mehmet Fatih Kucuk, Ayse Oz, Sultan Aydin

**Affiliations:** 1 Department of Ophthalmology, Antalya Training and Research Hospital, 07100 Antalya, Turkey; 2 Department of Pediatric Hematology and Oncology, Antalya Training and Research Hospital, 07100 Antalya, Turkey

**Keywords:** bevacizumab, arteriovenous malformations, pediatric, OCTA, optical coherence tomography angiography

## Abstract

**Background**: Systemic bevacizumab is increasingly used in selected pediatric patients with peripheral arteriovenous malformations (AVMs), particularly when interventional or surgical treatment is unsuitable. Although vascular endothelial growth factor (VEGF) contributes to physiological vascular integrity, the effects of systemic VEGF inhibition on the normal retinal microvasculature in children remain poorly understood. Optical coherence tomography angiography (OCTA) enables noninvasive assessment of retinal microvascular and structural changes. **Aim**: To evaluate retinal microvascular and thickness parameters using OCTA in pediatric patients with peripheral AVMs receiving systemic bevacizumab and to assess potential subclinical ocular effects. **Methods**: This cross-sectional case–control study included 60 eyes of 30 participants: 15 pediatric patients with peripheral AVMs treated with systemic bevacizumab for at least 6 months and 15 age- and sex-matched controls. All participants underwent comprehensive ophthalmologic examination and OCTA imaging. Superficial and deep capillary plexus vessel densities, radial peripapillary capillary plexus vessel density, foveal avascular zone parameters, fractal dimension, outer retinal and choriocapillaris flow areas, and retinal thickness were analyzed using mixed-design analysis of variance. **Results**: No significant differences were observed between groups in vessel densities, foveal avascular zone parameters, fractal dimension, flow areas, or total and outer retinal thickness (*p* > 0.05). Inner retinal foveal thickness was significantly lower in the bevacizumab group in both eyes (right: *p* = 0.044; left: *p* = 0.046). **Conclusions**: The lower inner retinal foveal thickness observed in the bevacizumab group represents a cross-sectional structural difference in uncertain clinical significance. Although experimental studies provide a biological rationale for investigating retinal changes during VEGF inhibition, they do not establish that the difference observed in our cohort was treatment-related. Pre-existing differences, underlying disease characteristics, residual confounding, and chance in the context of multiple comparisons remain possible explanations.

## 1. Introduction

Arteriovenous malformations (AVMs) are high-flow congenital vascular anomalies characterized by direct shunting between arteries and veins without an intervening capillary bed. In childhood, they most commonly involve regions such as the head and neck and the extremities. Their natural course is progressive, with a high tendency for recurrence; therefore, the management of pediatric AVMs requires a multidisciplinary and long-term approach [1,2,3]. Current management of pediatric peripheral AVMs consists of image-guided interventional procedures (e.g., nidus-level embolization and sclerotherapy), with surgical excision reserved for selected cases [4,5].

Recent advances in the molecular understanding of AVMs and other vascular anomalies have increased interest in targeted medical therapies. Anti-angiogenic strategies have gained particular attention due to their ability to directly modulate abnormal vascular growth and the inflammatory microenvironment associated with complex vascular anomalies [5,6,7].

Bevacizumab is a humanized immunoglobulin G1 monoclonal antibody that targets vascular endothelial growth factor (VEGF). It is widely used as a systemic anti-angiogenic agent in oncology and in the management of vascular anomalies and is commonly administered intravitreally in ophthalmology [7,8]. Experimental studies have shown that bevacizumab suppresses VEGF expression and angiogenesis in extracranial AVM cells while also influencing vascular stability and inflammatory signaling within the AVM microenvironment [6,7]. Although VEGF plays a critical role in embryonic vasculogenesis, it is also essential for maintaining vascular integrity, microvascular permeability, and organ function in adult tissues [9,10]. Experimental VEGF inhibition has been associated with capillary regression, loss of endothelial fenestrations, and impaired microvascular plasticity in adult vasculature [10,11].

Systemic bevacizumab therapy is associated with known adverse effects, including hypertension, proteinuria, bleeding, and thromboembolic events [11,12]. In ocular tissues, VEGF inhibition has been linked to choriocapillaris thinning, retinal pigment epithelium (RPE) damage, and photoreceptor loss in both experimental and clinical studies [13,14,15].

In pediatric oncology practice, bevacizumab is administered selectively in patients with peripheral AVMs who are unsuitable for interventional or surgical treatment or who demonstrate progressive disease despite prior therapies. At our center, bevacizumab is administered intravenously at a dose of 150 mg/m^2^ every 14 days.

The aim of this study was to evaluate the potential effects of systemic bevacizumab therapy on retinal vascular structures in pediatric patients with peripheral AVMs and to assess possible adverse ocular changes in otherwise healthy eyes.

## 2. Materials and Methods

### 2.1. Study Design and Participants

Participants in the bevacizumab group were recruited from pediatric patients with peripheral arteriovenous malformations (AVMs) receiving systemic bevacizumab and followed at the Department of Pediatric Hematology and Oncology, Antalya Training and Research Hospital, Antalya, Türkiye, between July 2025 and May 2026. Eligible patients were identified through [specify the participant selection method]. Ophthalmologic examinations and optical coherence tomography (OCT) and OCT angiography (OCTA) assessments were performed by the Department of Ophthalmology at the same institution. Age-matched healthy controls were recruited from [specify the source] during [specify the control recruitment period], according to the eligibility criteria described below.

Peripheral arteriovenous malformations were diagnosed through clinical assessment. The diagnosis was supported by imaging findings demonstrating MRG. Diagnostic findings were reviewed by a pediatric hematologist and a multidisciplinary team.

This prospective, cross-sectional case–control study included 60 eyes from 30 participants. The study group comprised 15 pediatric patients with peripheral arteriovenous malformations (AVMs) who had received systemic bevacizumab monotherapy for at least 6 months. The median recorded duration of bevacizumab therapy was 7.5 months (range, 6–14 months), with a median of 16.5 administrations (range, 12–28). No patients underwent concurrent sclerotherapy or surgery or received additional medical treatment during bevacizumab therapy before OCT/OCTA assessment. Fifteen age-matched healthy individuals served as the control group.

The study was approved by the Ethics Committee of Antalya Training and Research Hospital (approval date: 29 May 2025; approval number: 9/5) and conducted in accordance with the Declaration of Helsinki. Written informed consent was obtained from all participants and/or their legal guardians.

Participants aged 5–18 years with reliable OCTA imaging were included. Individuals with ocular or systemic conditions affecting the retina or choroid and those with a history of ocular surgery or trauma were excluded. Control participants had no systemic or ocular disease and a best-corrected visual acuity of 20/20.

### 2.2. Ophthalmologic Examination

All participants underwent a comprehensive ophthalmologic examination, including assessment of best-corrected visual acuity (BCVA), measurement of intraocular pressure using a non-contact tonometer, central corneal thickness measurement (pachymetry), slit-lamp biomicroscopy, and dilated fundus examination. OCT/OCTA image acquisition and evaluation were performed without masking to treatment status. Quantitative measurements were generated using the device’s automated analysis software.

### 2.3. Imaging Techniques

Optical coherence tomography angiography (OCTA) was performed using the AngioVue Imaging System (Optovue RTVue XR Avanti; Fremont, CA, USA). All scans were acquired by the same examiner between 08:30 a.m. and 12:30 p.m. Images with a signal strength index <6, motion artifacts, or segmentation errors were excluded.

Macular imaging was obtained using the HD Angio Retina 6 mm × 6 mm protocol. Automated software analysis provided vessel density measurements of the superficial and deep capillary plexuses (SCP and DCP, respectively) for the whole image and predefined fovea-centered macular regions, as well as the foveal avascular zone (FAZ) area and non-flow area and flow areas of the outer retina and choriocapillaris. Central foveal thickness was measured using the retinal map mode.

Peripapillary vascular assessment was performed using a 4.5 mm × 4.5 mm scan centered on the optic disc. Vessel density of the radial peripapillary capillary (RPC) plexus was calculated for the superior, inferior, nasal, and temporal sectors within the retinal nerve fiber layer.

### 2.4. Statistical Analysis

Statistical analyses were performed using IBM SPSS Statistics for Windows, version 27 (IBM Corp., Armonk, NY, USA). Descriptive statistics were expressed as frequencies and percentages, means ± standard deviations, or medians (minimum–maximum), as appropriate. Normality was assessed using the Shapiro–Wilk test.

Each retinal outcome was analyzed using mixed-design analysis of variance, with group (bevacizumab-treated patients versus controls) as the between-participant factor and eye (right versus left) as the within-participant repeated-measures factor. The model included group, eye, and their interaction to account for the paired nature of bilateral measurements. Participants, rather than individual eyes, constituted the independent sampling units. Because the within-participant factor had only two levels, no sphericity correction was required.

To address multiplicity across retinal outcomes, the 58 eye-specific between-group comparisons covering the 29 parameters presented in Table 1, Table 2, Table 3, Table 4 and Table 5 were treated as a single comparison family. Bonferroni-adjusted *p*-values were calculated by multiplying each unadjusted *p*-value by 58, with values capped at 1.00. Both unadjusted and adjusted *p*-values were reported, and an adjusted *p*-value < 0.05 was considered statistically significant. Within-group comparisons between right and left eyes were considered exploratory and were not included in this between-group comparison family.

## 3. Results

A total of 60 eyes from 30 participants were included in the study: 30 eyes of 15 pediatric patients with peripheral AVMs receiving systemic bevacizumab therapy and 30 eyes of 15 age- and sex-matched healthy controls. There were no statistically significant differences between the groups in terms of age or sex distribution.

The available OCTA dataset included 15 bevacizumab-treated patients and 15 controls. The mean age was 11.92 ± 4.48 years in the bevacizumab group and 11.62 ± 4.50 years in the control group. Each group comprised six females (46.2%) and seven males (53.8%). Mean intraocular pressure in the right and left eyes was 15.42 ± 2.67 and 14.89 ± 2.83 mmHg, respectively, in the bevacizumab group, compared with 15.54 ± 3.56 and 15.72 ± 3.48 mmHg in controls. Mean central corneal thickness in the right and left eyes was 534.62 ± 27.22 and 535.92 ± 30.08 µm, respectively, in the bevacizumab group, compared with 563.15 ± 29.23 and 568.08 ± 29.23 µm in controls.

No statistically significant differences were observed between the control and bevacizumab groups in small-vessel density measurements or other RPC plexus parameters evaluated across the whole image, inside-disc, and peripapillary regions (*p* > 0.05 for all) (Table 1).

No statistically significant differences were found between the control and bevacizumab groups in whole retinal, foveal, parafoveal, or perifoveal vessel density measurements of either the SCP or DCP (*p* > 0.05 for all) (Table 2 and Table 3).

There were no statistically significant differences between the control and bevacizumab groups in FAZ area, FAZ perimeter, fractal dimension, outer retinal flow area, choriocapillaris flow area, or non-flow area in the SCP for either eye (*p* > 0.05 for all) (Table 4).

No statistically significant differences were found between the groups in total retinal thickness measurements (foveal, parafoveal, and perifoveal) or outer retinal layer thicknesses (*p* > 0.05 for all). In contrast, inner retinal foveal thickness measurements showed statistically significant differences between the groups in both eyes. Inner retinal foveal thickness was lower in the bevacizumab group than in controls, with nominally significant unadjusted *p*-values for the right and left eyes (*p* = 0.044 and *p* = 0.046, respectively) (Figure 1). No significant differences were observed between the groups in inner retinal parafoveal or perifoveal thickness measurements (Table 5).

## 4. Discussion

In this study, we evaluated retinal microvascular and retinal thickness parameters using OCTA in healthy eyes of pediatric patients with peripheral AVMs receiving systemic bevacizumab therapy. Our findings demonstrated a limited but statistically significant reduction in inner retinal foveal thickness in both the right and left eyes of the bevacizumab group compared with the control group. In contrast, no significant differences were observed in RPC plexus vessel density, SCP and DCP vessel densities, FAZ area, FAZ perimeter, fractal dimension, or outer retinal or choriocapillaris flow areas. These results suggest that systemic bevacizumab therapy was not associated with widespread or pronounced structural impairment of the retinal microcirculation in pediatric patients but may induce limited, subclinical effects at the level of the inner retina.

### 4.1. Retinal Microvascular Densities and the Systemic Anti-VEGF Effect

In our study, preservation of vessel densities in the SCP and DCP indicates that systemic bevacizumab therapy does not cause a significant hemodynamic disturbance in the retinal microcirculation of pediatric patients. The absence of significant differences in FAZ area and FAZ perimeter further supports the preservation of macular microvascular integrity in this population. These findings are consistent with current concepts of AVM pathogenesis. It has been reported that increased VEGF expression and an inflammatory microenvironment in AVMs predominantly exert their effects locally at the nidus level, while physiological vascular beds are relatively preserved [7].

Similarly, clinical studies investigating systemic bevacizumab use in hereditary hemorrhagic telangiectasia and brain AVMs have not reported significant vascular adverse events [16,17]. Long-term data suggest that systemic bevacizumab may exert selective effects on pathological vascular beds while having limited impact on normal vascular structures [18]. A preferential effect of systemic bevacizumab on pathological rather than physiological vascular beds remains a hypothesis. Our study was not designed to test vascular selectivity, and the absence of statistically significant differences in retinal vascular parameters does not establish preservation of normal vascular beds or preferential activity within AVMs. This hypothesis requires investigation in studies directly assessing both pathological and physiological vascular beds before and during treatment.

### 4.2. Possible Biological Significance of Decreased Inner Retinal Foveal Thickness

The most notable finding of our study was the reduction in inner retinal foveal thickness in the bevacizumab group. Given the cross-sectional design of the study, this finding should be interpreted as an association rather than evidence of a causal effect of systemic bevacizumab therapy.

Both in vivo studies and experimental animal models have shown that VEGF not only plays a role in pathological and physiological angiogenesis but also exerts neuroprotective effects on retinal neuronal survival. Using a retinal ischemia–reperfusion model, Nishijima et al. demonstrated that VEGF-A acted as an endogenous survival factor for retinal neurons and that inhibition of VEGF signaling increased neuronal cell loss [19]. Similarly, Brar et al. reported that VEGF protected retinal ganglion cells against oxidative stress, an effect that could be blocked by bevacizumab [20].

Taken together, these findings suggest that increased neuronal vulnerability due to VEGF inhibition may result in structural thinning of the inner retinal layers, providing a plausible biological mechanism for the observed reduction in inner retinal foveal thickness. Supporting this interpretation, animal studies have reported that VEGF inhibition may be associated with thinning of the choriocapillaris and photoreceptor degeneration [15]. In addition, in vitro studies have demonstrated that bevacizumab can impair barrier function and cellular integrity in RPE cells [16,21]. However, these findings are largely derived from experimental models, and their direct translation to the human retina under clinical dosing and systemic administration remains limited and variable. Importantly, the observed structural difference in the present study was not accompanied by any detectable visual symptoms or abnormalities on routine ophthalmologic examination, suggesting that the finding is subclinical at the time of assessment. Nevertheless, the absence of widespread retinal thinning or loss of vascular density suggests that the observed reduction in inner retinal foveal thickness may represent an early, limited, and subclinical effect. Therefore, these findings should be interpreted with caution and without implying causality.

### 4.3. Peripapillary Microvascular Findings Related to the Optic Nerve Head

In this study, no statistically significant differences in RPC plexus vessel density were detected between the bevacizumab and control groups. Given the cross-sectional design and small sample size, this finding should not be interpreted as evidence that bevacizumab has no effect on peripapillary microvascular circulation.

Systemic bevacizumab has emerged as an effective therapeutic option for bleeding control and symptomatic improvement in hemorrhagic telangiectasia and complex AVMs [6,14,17]. However, data regarding the ocular safety profile of systemic anti-VEGF therapies in pediatric patients with peripheral AVMs remain extremely limited. To our knowledge, data on OCTA-based retinal microvascular assessment in children receiving systemic bevacizumab for peripheral AVMs remain very limited.

### 4.4. Limitations

This study has several limitations. First, the small sample size of 15 treated patients and 15 controls limits statistical power and increases the risk of type II error; therefore, potentially clinically meaningful differences in OCTA parameters may have remained undetected. Although both eyes were evaluated, within-participant correlation limits the additional information provided by bilateral measurements. Consequently, nonsignificant findings should not be interpreted as evidence of equivalence between groups or confirmation of ocular safety. The evaluation of numerous OCTA parameters also raises concerns regarding multiple testing and warrants cautious interpretation of the isolated finding of lower inner retinal foveal thickness. Second, the cross-sectional design and absence of pretreatment OCT/OCTA measurements preclude assessment of changes over time and prevent causal attribution of the observed differences to systemic bevacizumab therapy. Third, the lack of long-term follow-up limits assessment of the persistence, progression, and clinical significance of these findings. Finally, the absence of functional retinal assessments, such as microperimetry or electrophysiological testing, limits the evaluation of potential relationships between structural differences and retinal function. These findings should therefore be considered exploratory. Larger, adequately powered prospective studies incorporating pretreatment and serial OCT/OCTA measurements, predefined primary outcomes, and long-term functional follow-up are needed to clarify these associations.

## 5. Conclusions

In this cross-sectional study, no statistically significant differences were detected in retinal vascular densities or most structural parameters between pediatric patients with peripheral AVMs receiving systemic bevacizumab and healthy controls. However, the small sample size limits the ability to exclude clinically meaningful retinal differences. Lower inner retinal foveal thickness was observed in the treated group, but the cross-sectional design and absence of pretreatment OCT/OCTA measurements prevent attribution of this finding to bevacizumab therapy. Its clinical significance remains uncertain, and it should be considered exploratory. Continued ophthalmologic assessment during treatment and larger prospective studies incorporating pretreatment and serial OCT/OCTA measurements, functional evaluations, and long-term follow-up would help clarify these observations and better characterize the ocular safety profile of systemic bevacizumab in pediatric populations.

## Figures and Tables

**Figure 1:**
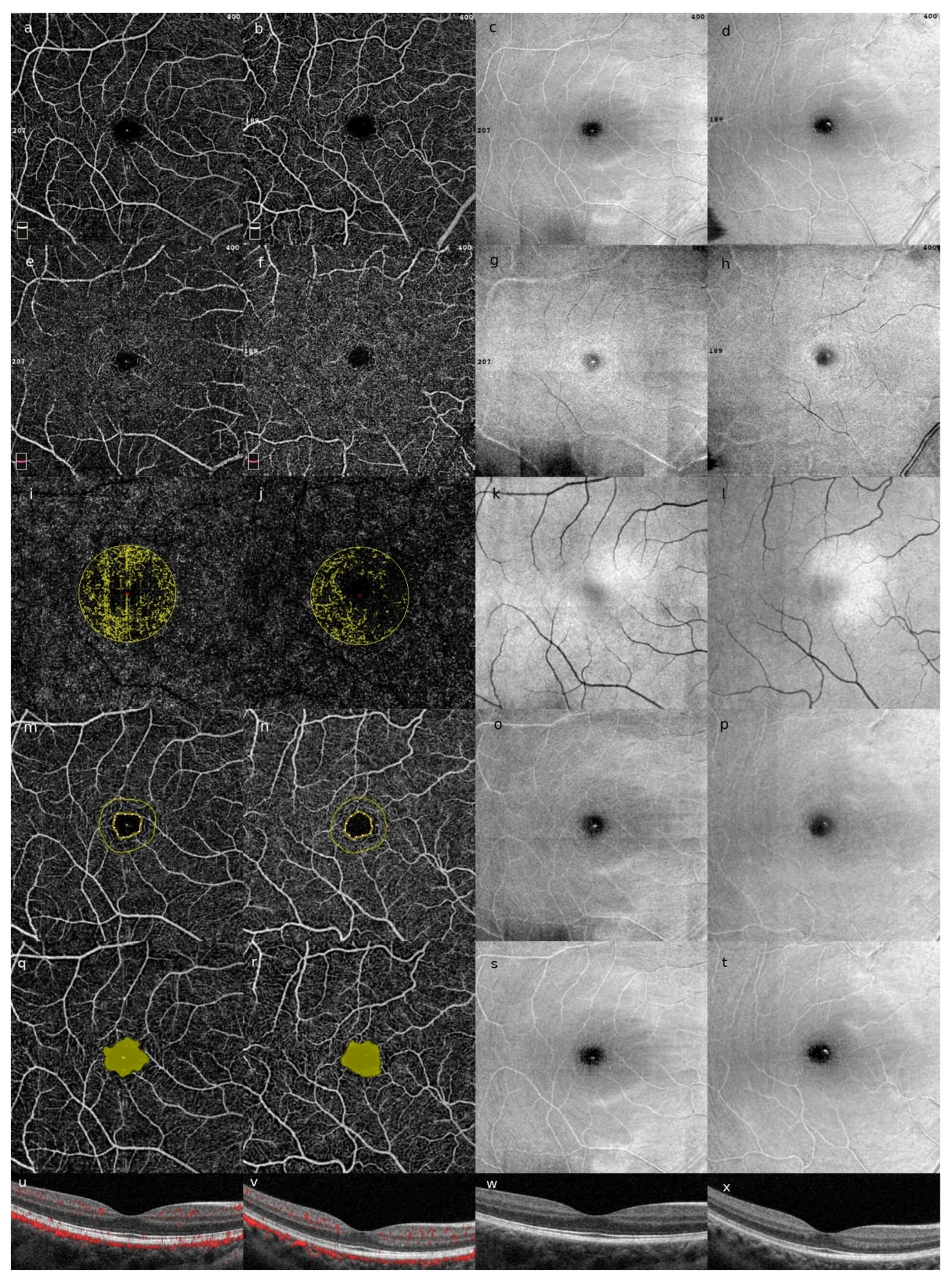
OCTA images of the macular region in a 14-year-old patient receiving systemic bevacizumab (first and third columns) and a healthy control (second and fourth columns). Superficial capillary plexus (**a**,**b**), deep capillary plexus (**e**,**f**), outer retina flow area (**i**,**j**), foveal avascular zone (**m**,**n**), and non-flow area (**q**,**r**) measurements were comparable between groups. En face images of the corresponding layers (**c**,**d**,**g**,**h**,**k**,**l**,**o**,**p**,**s**,**t**) similarly demonstrate preserved macular microvascular architecture, with no statistically significant differences observed. Cross-sectional OCT B-scans (**u**–**x**) show reduced inner retinal thickness in the patient treated with bevacizumab. The small rectangles in the lower left corner of (**a**,**b**,**e**,**f**) are device-generated icons indicating the depth of the segmented slab (superficial or deep capillary plexus). In (**i**,**j**), yellow pixels indicate the detected flow signal within the yellow circular measurement area; in (**m**,**n**), the inner yellow outline delineates the FAZ boundary and the outer yellow ring delineates the 300 µm-wide band surrounding the FAZ, as automatically drawn by the software; and in (**q**,**r**), the yellow-filled region represents the non-flow area. In (**u**,**v**), red indicates the OCTA flow signal overlaid on the B-scans.

**Table 1:** RPC vascular density values and their comparison between the study groups.

	Group		Test Statistics ^†^		
	Control	Bevacizumab	F	*p*	η^2^
	*n* = 15	*n* = 15			
Small Vessel Density, Whole Disc (%)					
Right	49.59 ± 2.43	49.41 ± 1.61	0.057	0.813	0.002
Left	49.68 ± 1.99	50.25 ± 1.77	0.676	0.418	0.024
Test statistics ^¥^	F = 0.042; *p* = 0.840; η^2^ = 0.001	F = 3.379; *p* = 0.077; η^2^ = 0.108			
	F = 0.050; *p* = 0.824; η^2^ = 0.002				
Small Vessel Density, Inside Disc (%)					
Right	51.57 ± 4.92	50.43 ± 4.5	0.438	0.514	0.015
Left	51.61 ± 4	50.84 ± 4.64	0.239	0.629	0.008
Test statistics ^¥^	F = 0.001; *p* = 0.970; η^2^ = 0.001	F = 0.153; *p* = 0.698; η^2^ = 0.005			
	F = 0.416; *p* = 0.524; η^2^ = 0.015				
Small Vessel Density, Peripapillary (%)					
Right	51.63 ± 2.79	50.83 ± 2.19	0.762	0.390	0.026
Left	51.79 ± 2.26	52.03 ± 2.36	0.081	0.778	0.003
Test statistics ^¥^	F = 0.074; *p* = 0.787; η^2^ = 0.003	F = 4.493; *p* = 0.043; η^2^ = 0.138			
	F = 17,214.692; *p* = 0.001; η^2^ = 0.998				
All Vessel Density, Whole Disc (%)					
Right	56.28 ± 2.11	55.93 ± 1.73	0.252	0.620	0.009
Left	56.19 ± 2	56.43 ± 1.71	0.125	0.727	0.004
Test Statistics ^¥^	F = 0.045; *p* = 0.833; η^2^ = 0.002	F = 1.296; *p* = 0.265; η^2^ = 0.044			
	F = 0.008; *p* = 0.928; η^2^ = 0.001				
All Vessel Density, Inside Disc (%)					
Right	61.21 ± 4.07	61.1 ± 3.89	0.006	0.938	0.001
Left	61.77 ± 3.05	61.12 ± 3.9	0.256	0.617	0.009
Test statistics ^¥^	F = 0.358; *p* = 0.554; η^2^ = 0.013	F = 0.001; *p* = 0.983; η^2^ = 0.001			
	F = 0.100; *p* = 0.754; η^2^ = 0.004				
All Vessel Density, Peripapillary (%)					
Right	58.3 ± 2.36	57.21 ± 2.09	1.805	0.190	0.061
Left	58.15 ± 2.19	58.03 ± 1.92	0.023	0.881	0.001
Test statistics ^¥^	F = 0.112; *p* = 0.740; η^2^ = 0.004	F = 3.267; *p* = 0.081; η^2^ = 0.104			
	F = 0.716; *p* = 0.405; η^2^ = 0.025				

RPC: radial peripapillary capillary, F: mixed-design analysis of variance, η^2^: effect size. ^¥^ within-group comparison, ^†^ between-group comparison. Values are presented as mean ± standard deviation.

**Table 2:** Regional vascular density values of the SCP and comparison between the study groups.

	Groups		Test Statistics ^†^		
	Control	Bevacizumab	F	*p*	η^2^
	*n* = 15	*n* = 15			
Whole (%)					
Right	50.31 ± 3.38	49.45 ± 2.36	0.653	0.426	0.023
Left	48.88 ± 2.32	48.85 ± 3.82	0.001	0.977	0.001
Test statistics ^¥^	F = 3.821; *p* = 0.061; η^2^ = 0.120	F = 0.676; *p* = 0.418; η^2^ = 0.024			
	F = 0.207; *p* = 0.653; η^2^ = 0.007				
Foveal (%)					
Right	23.41 ± 6.31	21.19 ± 6.42	0.913	0.348	0.032
Left	23.64 ± 6.57	20.13 ± 6.3	2.228	0.147	0.074
Test statistics ^¥^	F = 0.142; *p* = 0.710; η^2^ = 0.005	F = 2.885; *p* = 0.101; η^2^ = 0.093			
	F = 1.556; *p* = 0.223; η^2^ = 0.053				
Parafoveal (%)					
Right	52.63 ± 3.98	51.91 ± 2.49	0.353	0.557	0.012
Left	50.87 ± 3.94	51.41 ± 4.95	0.109	0.743	0.004
Test statistics ^¥^	F = 2.842; *p* = 0.103; η^2^ = 0.092	F = 0.225; *p* = 0.639; η^2^ = 0.008			
	F = 0.005; *p* = 0.942; η^2^ = 0.001				
Perifoveal (%)					
Right	50.51 ± 3.54	49.91 ± 2.51	0.287	0.596	0.010
Left	49.17 ± 2.75	49.33 ± 3.94	0.018	0.894	0.001
Test statistics ^¥^	F = 3.111; *p* = 0.089; η^2^ = 0.100	F = 0.570; *p* = 0.457; η^2^ = 0.020			
	F = 0.042; *p* = 0.838; η^2^ = 0.002				

SCP: superficial capillary plexus, F: mixed-design analysis of variance, η^2^: effect size. ^¥^ within-group comparison, ^†^ between-group comparison. Values are presented as mean ± standard deviation.

**Table 3:** Regional vascular density values of the DCP and their comparison between the study groups.

	Groups		Test Statistics ^†^		
	Control	Bevacizumab	F	*p*	η^2^
	*n* = 15	*n* = 15			
Whole (%)					
Right	50.92 ± 5.43	49.53 ± 4.68	0.560	0.460	0.020
Left	50.17 ± 5.58	47.94 ± 5.56	1.205	0.282	0.041
Test statistics ^¥^	F = 0.451; *p* = 0.508; η^2^ = 0.016	F = 2.052; *p* = 0.163; η^2^ = 0.068			
	F = 1.035; *p* = 0.318; η^2^ = 0.036				
Foveal (%)					
Right	40.5 ± 5.71	39.31 ± 7.19	0.253	0.619	0.009
Left	41.24 ± 7.13	37.82 ± 7.43	1.653	0.209	0.056
Test statistics ^¥^	F = 0.764; *p* = 0.390; η^2^ = 0.027	F = 3.082; *p* = 0.090; η^2^ = 0.099			
	F = 0.888; *p* = 0.354; η^2^ = 0.031				
Parafoveal (%)					
Right	55.91 ± 4.77	54.94 ± 3.97	0.364	0.551	0.013
Left	55.27 ± 4.59	54.29 ± 4.43	0.349	0.560	0.012
Test statistics ^¥^	F = 0.446; *p* = 0.510; η^2^ = 0.016	F = 0.456; *p* = 0.505; η^2^ = 0.016			
	F = 0.431; *p* = 0.517; η^2^ = 0.015				
Perifoveal (%)					
Right	52.51 ± 5.81	50.95 ± 5.42	0.578	0.453	0.020
Left	51.48 ± 6.12	48.67 ± 6.14	1.572	0.220	0.053
Test statistics ^¥^	F = 0.736; *p* = 0.398; η^2^ = 0.026	F = 3.609; *p* = 0.068; η^2^ = 0.114			
	F = 1.224; *p* = 0.278; η^2^ = 0.042				

DCP: deep capillary plexus, F: mixed-design analysis of variance, η^2^: effect size. ^¥^ within-group comparison, ^†^ between-group comparison. Values are presented as mean ± standard deviation.

**Table 4:** Comparison of the FAZ area and perimeter, FD, outer retinal flow area, choriocapillaris flow area, and non-flow area in the SCP between the study groups.

	Group		Test Statistics ^†^		
	Control	Bevacizumab	F	*p*	η^2^
	*n* = 15	*n* = 15			
FAZ (mm^2^)					
Right	0.23 ± 0.09	0.26 ± 0.1	0.858	0.362	0.030
Left	0.23 ± 0.09	0.27 ± 0.1	1.332	0.258	0.045
Test statistics ^¥^	F = 1.043; *p* = 0.316; η^2^ = 0.036	F = 0.010; *p* = 0.919; η^2^ = 0.001			
	F = 1.108; *p* = 0.302; η^2^ = 0.038				
FAZ Perimeter					
Right	1.83 ± 0.37	1.96 ± 0.36	1.009	0.324	0.035
Left	1.8 ± 0.38	1.98 ± 0.38	1.631	0.212	0.055
Test statistics ^¥^	F = 0.607; *p* = 0.443; η^2^ = 0.021	F = 0.157; *p* = 0.695; η^2^ = 0.006			
	F = 1.359; *p* = 0.254; η^2^ = 0.046				
FD					
Right	54.42 ± 4.55	55.49 ± 2.77	0.611	0.441	0.021
Left	53.96 ± 5.03	53.68 ± 4.8	0.024	0.878	0.001
Test statistics ^¥^	F = 0.216; *p* = 0.646; η^2^ = 0.008	F = 3.396; *p* = 0.076; η^2^ = 0.108			
	F = 0.076; *p* = 0.784; η^2^ = 0.003				
Outer Retinal Flow Area (mm^2^)					
Right	0.76 ± 0.3	0.88 ± 0.39	0.888	0.354	0.031
Left	0.86 ± 0.33	0.94 ± 0.44	0.339	0.565	0.012
Test statistics ^¥^	F = 1.135; *p* = 0.296; η^2^ = 0.039	F = 0.466; *p* = 0.501; η^2^ = 0.016			
	F = 0.758; *p* = 0.391; η^2^ = 0.026				
Choriocapillaris Flow Area (mm^2^)					
Right	2.23 ± 0.09	2.19 ± 0.09	1.994	0.169	0.066
Left	2.21 ± 0.1	2.18 ± 0.12	0.615	0.440	0.021
Test statistics ^¥^	F = 1.449; *p* = 0.239; η^2^ = 0.049	F = 0.234; *p* = 0.633; η^2^ = 0.008			
	F = 0.408; *p* = 0.529; η^2^ = 0.017				
Non-Flow Area in SCP (mm^2^)					
Right	0.44 ± 0.11	0.49 ± 0.11	1.488	0.233	0.050
Left	0.44 ± 0.11	0.5 ± 0.14	1.587	0.218	0.054
Test statistics^¥^	F = 0.001; *p* = 0.972; η^2^ = 0.001	F = 0.263; *p* = 0.612; η^2^ = 0.009			
	F = 1.635; *p* = 0.212; η^2^ = 0.055				

FAZ: foveal avascular zone, FD: fractal dimension, SCP: superficial capillary plexus, F: mixed-design analysis of variance, η^2^: effect size. ^¥^ within-group comparison, ^†^ between-group comparison. Values are presented as mean ± standard deviation.

**Table 5:** Regional mean retinal thickness values and their comparison between the study groups.

	Group		Test Statistics ^†^		
	Control	Bevacizumab	F	*p*	η^2^
	*n* = 15	*n* = 15			
Full Retinal Foveal Thickness (µm)					
Right	254.13 ± 18.02	250.4 ± 16.95	0.342	0.564	0.012
Left	254.33 ± 18.2	249.6 ± 19.97	0.460	0.503	0.016
Test statistics ^¥^	F = 0.007; *p* = 0.932; η^2^ = 0.001	F = 0.119; *p* = 0.732; η^2^ = 0.004			
	F = 0.426; *p* = 0.519; η^2^ = 0.015				
Full Retinal Parafoveal Thickness (µm)					
Right	313.27 ± 12.91	317.8 ± 17.16	0.668	0.412	0.023
Left	315.47 ± 14.82	315.8 ± 17.59	0.003	0.956	0.001
Test statistics ^¥^	F = 0.533; *p* = 0.471; η^2^ = 0.019	F = 0.440; *p* = 0.512; η^2^ = 0.015			
	F = 0.208; *p* = 0.652; η^2^ = 0.007				
Full Retinal Perifoveal Thickness (µm)					
Right	285.47 ± 13.45	290 ± 16.63	0.674	0.419	0.024
Left	289 ± 15.83	287.8 ± 14.46	0.047	0.830	0.002
Test statistics ^¥^	F = 3.269; *p* = 0.081; η^2^ = 0.105	F = 1.267; *p* = 0.270; η^2^ = 0.043			
	F = 0.097; *p* = 0.758; η^2^ = 0.003				
Inner Retinal Foveal Thickness					
Right	76.54 ± 9.28	69.38 ± 8.69	4.115	0.044	0.146
Left	74.54 ± 12.24	66.38 ± 8.02	4.038	0.046	0.144
Test statistics ^¥^	F = 2.245; *p* = 0.147; η^2^ = 0.086	F = 5.050; *p* = 0.034; η^2^ = 0.174			
	F = 1517.012; *p* = 0.001; η^2^ = 0.984				
Inner Retinal Parafoveal Thickness					
Right	129.53 ± 8.27	129.93 ± 9.2	0.016	0.901	0.001
Left	131.6 ± 7.54	128.4 ± 9.92	0.990	0.328	0.034
Test statistics ^¥^	F = 1.369; *p* = 0.252; η^2^ = 0.047	F = 0.753; *p* = 0.393; η^2^ = 0.026			
	F = 0.225; *p* = 0.639; η^2^ = 0.008				
Inner Retinal Perifoveal Thickness					
Right	112.53 ± 7.7	112.07 ± 9.07	0.023	0.880	0.001
Left	114 ± 7.97	111.73 ± 8.55	0.564	0.459	0.020
Test statistics ^¥^	F = 1.651; *p* = 0.209; η^2^ = 0.056	F = 0.085; *p* = 0.772; η^2^ = 0.003			
	F = 0.217; *p* = 0.645; η^2^ = 0.008				
Outer Retinal Foveal Thickness					
Right	179.13 ± 10.71	178.93 ± 10.48	0.003	0.959	0.001
Left	180.8 ± 8.91	181.27 ± 16.03	0.010	0.922	0.001
Test statistics ^¥^	F = 0.486; *p* = 0.492; η^2^ = 0.017	F = 0.952; *p* = 0.338; η^2^ = 0.033			
	F = 0.001; *p* = 0.974; η^2^ = 0.001				
Outer Retinal Parafoveal Thickness					
Right	183.87 ± 9.6	187.8 ± 10.82	1.109	0.301	0.038
Left	183.8 ± 9.34	187.33 ± 13.2	0.716	0.405	0.025
Test statistics ^¥^	F = 0.001; *p* = 0.981; η^2^ = 0.001	F = 0.027; *p* = 0.870; η^2^ = 0.001			
	F = 1.194; *p* = 0.284; η^2^ = 0.041				
Outer Retinal Perifoveal Thickness					
Right	172.73 ± 7.81	177.8 ± 10.02	2.386	0.134	0.079
Left	175.13 ± 8.8	176.13 ± 9.2	0.093	0.763	0.003
Test statistics ^¥^	F = 1.693; *p* = 0.204; η^2^ = 0.057	F = 0.816; *p* = 0.374; η^2^ = 0.028			
	F = 1.014; *p* = 0.322; η^2^ = 0.035				

F: mixed-design analysis of variance, η^2^: effect size. ^¥^ within-group comparison, ^†^ between-group comparison. Values are presented as mean ± standard deviation.

## Data Availability

The datasets generated and/or analyzed in the current study are available from the corresponding author upon reasonable request.

## Abbreviations

BCVA	Best-Corrected Visual Acuity
IOP	Intraocular Pressure
CCT	Central Corneal Thickness
AVM	Arteriovenous Malformation
OCT	Optical Coherence Tomography
OCTA	Optical Coherence Tomography Angiography
VEGF	Vascular Endothelial Growth Factor
RPE	Retinal Pigment Epithelium
SCP	Superficial Capillary Plexuses
DCP	Deep Capillary Plexuses
FAZ	Foveal Avascular Zone
RPC	Radial Peripapillary Capillary
